# Arborinol methyl ether from *Areca catechu* L.

**DOI:** 10.1107/S1600536810030758

**Published:** 2010-08-11

**Authors:** Xixin He, Yajun Li, Cuixian Zhang, Xiaopeng Hu

**Affiliations:** a College of Chinese Materia Medica, Guangzhou University of Chinese Medicine, Guangzhou 510006, People’s Republic of China; b School of Pharmaceutical Science, Sun Yat-sen University, Guangzhou 510006, People’s Republic of China

## Abstract

The title compound isolated from *Areca catechu* L. (common name: arborinol methyl ether; a member of the arborane family) was established as 3α-methoxyarbor-9(11)-ene, C_31_H_52_O. Rings *A*/*B*/*C*/*D* assume a chair conformation, while ring *E* has an envelope conformation. The absolute configuration was determined to be (3*R*,5*R*,8*S*,10*S*,13*R*,14*S*,17*S*,18*S*, 21*S*) by analysis of Bijvoet pairs based on resonant scattering of light atoms, yielding a Hooft parameter *y* of −0.03 (3).

## Related literature

For the biological activity of *Areca catechu* L. compounds, see: Dar *et al.* (1997 ▶); Hocart & Fankhauser (1996 ▶); Iwamoto *et al.* (1988 ▶); Kusumoto *et al.* (1995 ▶); Norton (1998 ▶); Lee & Choi (1999 ▶); Ohmoto & Natori (1969 ▶); Chan *et al.* (2008 ▶); Pithayanukul *et al.* (2009 ▶); Zhang *et al.* (2010 ▶). For related structures, see: Corrêa *et al.* (2009 ▶); Khera *et al.* (2003 ▶); Takahashi & Iitaka (1972 ▶). Analysis of the absolute configuration was performed by using likelihood methods (Hooft *et al.*, 2008 ▶) using *PLATON* (Spek, 2009 ▶).
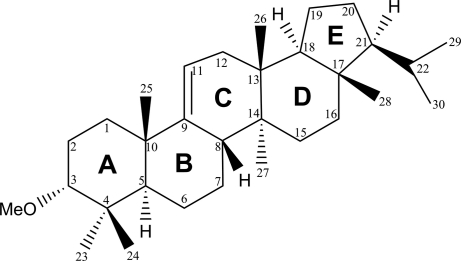


## Experimental

### 

#### Crystal data

C_31_H_52_O
*M* *_r_* = 440.73
Triclinic, 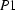

*a* = 6.2684 (2) Å
*b* = 7.1162 (3) Å
*c* = 16.0814 (5) Å
α = 96.812 (3)°
β = 91.079 (3)°
γ = 114.397 (4)°
*V* = 646.86 (4) Å^3^
*Z* = 1
Cu *K*α radiation
μ = 0.48 mm^−1^
*T* = 120 K
0.60 × 0.50 × 0.40 mm

#### Data collection

Oxford Diffraction Xcalibur Eos Gemini diffractometer
Absorption correction: multi-scan (*CrysAlis PRO*; Oxford Diffraction, 2010 ▶) *T*_min_ = 0.658, *T*_max_ = 1.0
10247 measured reflections
4415 independent reflections
4408 reflections with *I* > 2σ(*I*)
*R*_int_ = 0.011

#### Refinement

*R*[*F* ^2^ > 2σ(*F* ^2^)] = 0.036
*wR*(*F* ^2^) = 0.096
*S* = 1.04
4415 reflections
298 parameters
3 restraints
H-atom parameters constrained
Δρ_max_ = 0.27 e Å^−3^
Δρ_min_ = −0.18 e Å^−3^
Absolute structure: Flack (1983 ▶), 1952 Friedel pairs
Flack parameter: 0.02 (22)

### 

Data collection: *CrysAlis PRO* (Oxford Diffraction, 2010 ▶); cell refinement: *CrysAlis PRO*; data reduction: *CrysAlis PRO*; program(s) used to solve structure: *SHELXS97* (Sheldrick, 2008 ▶); program(s) used to refine structure: *SHELXL97* (Sheldrick, 2008 ▶); molecular graphics: *SHELXTL* (Sheldrick, 2008 ▶); software used to prepare material for publication: *SHELXL97* and *publCIF* (Westrip, 2010 ▶).

## Supplementary Material

Crystal structure: contains datablocks I, global. DOI: 10.1107/S1600536810030758/si2282sup1.cif

Structure factors: contains datablocks I. DOI: 10.1107/S1600536810030758/si2282Isup2.hkl

Additional supplementary materials:  crystallographic information; 3D view; checkCIF report

## Figures and Tables

**Fig. 1.**
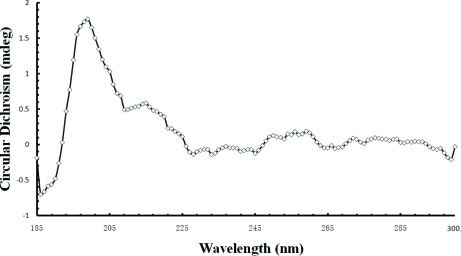
Circular Dichroism Spectroscopy of (I), measured on Chirascan Circular Dichroism Spectrometer, Applied PhotoPhysics (UK).

**Fig. 2.**
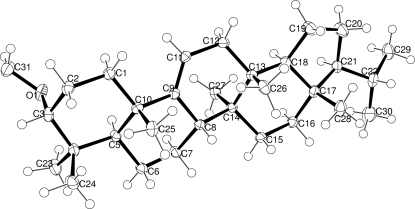
A view of the molecular structure of compound (I). The displacement ellipsoids are at the 50% probability level and H atoms are shown as small spheres of arbitrary radii..

## supplementary crystallographic information

### Comment

*Areca catechu* L. is an important economical plant in tropical and subtropical areas. Its ripe fruit is widely used in traditional Chinese medicine for treatment of constipation, oedema, beriberi and dyspepsia. Pharmacological research have shown areca nut possesses psychoactive (Hocart & Fankhauser, 1996, Norton, 1998), anti-depressant (Dar *et al.* 1997), anti-HIV-1 (Kusumoto *et al.*, 1995), anti-melanogenesis (Lee & Choi, 1999), anti-inflammatory (Pithayanukul *et al.*, 2009), anti-oxidant (Chan *et al.*, 2008), anti-tumor (Iwamoto *et al.*, 1988) and cytotoxic activities (Zhang *et al.* 2010). During our investigation of the anti-depressant activity of *Areca catechu L*., the title compound (I) was isolated from chloroform extract of areca nut.

The structure of (I) was analysed by spectroscopic and spectrometric analysis and proved to be arborinol methyl ether. The same compound has previously been found in species of Gramineous (Ohmoto & Natori 1969) and the structures of related compounds have been previously reported (Takahashi & Iitaka, 1972, Khera *et al.*, 2003, Corrêa *et al.*, 2009), their stereochemistry were specified by biosynthesis. In this study, X-ray crystallographic analysis of (I) was undertaken to establish the structure and to assign the absolute stereochemistry. The Flack parameter (Flack, 1983) *x* = 0.02 (22) is slightly ambiguous based on resonant scattering of the light atoms (the heaviest atom in this compound is oxygen). Thus analysis of the absolute configuration was further performed by using likelihood methods (Hooft *et al.*, 2008) with *PLATON* (Spek, 2009). The resulting value is *y* = -0.03 (3), corresponding to a probability P2(true) = 1.000 for this structure, confirming the absolute configuration. This value also agrees with the CD spectroscopic measurement result (Fig. 1). As shown in Fig. 2, rings A, B, C and D assume a chair conformation, while ring E adopts a envelope conformation. The A/B, C/D, and D/E ring junctions are *trans* fused about the C5DC10, C13DC14 and C17DC18 bonds, respectively. The absolute configuration of each chiral atom is 3*R*, 5*R*, 8*S*, 10*S*, 13*R*, 14*S*, 17*S*, 18*S*, 21*S* respectively. Compared with (I), the absolute configuration at all chiral centers of lupeol methyl ether is 3*S*, 5*R*, 8*R*, 9*S*,10*R*, 13*R*, 14*R*, 17*R*, 19*R*, while Fernane is 3*R*, 5*S*, 9*R*, 10*S*, 13*S*, 14*S*, 17*R*, 18*R*, 21*R*, agreeing well with the original results (Corrêa, *et al.*, 2009, Khera *et al.*, 2003).

### Experimental

The chloroform extract of dried areca nut was chromatographed on a silica gel (200–300 mesh) column with increasing concentrations of EtOAc in petroleum ether. The fractions eluting with petroleum ether were collected to afford crude compound. The pure title compound was obtained by recrystallization with chloroform. Single crystals were obtained by slow evaporation of chloroform at room temperature.

Regarding to the ambiguous Flack parameter *x* = 0.02 (22), TWIN/BASF instructions were tested  in a *parallel* refinement and resulted a BASF parameter of 0.02271, thus the single crystal used in data collection is barely a racemic mixture.

The title compound was a colorless crystal with mp 284~296 °C, [α]^20^_D_= +9.1° (c 0.01, CHCl3). 1H NMR (400 MHz, CDCl3) δ 5.22 (1*H*, d, J= 6.2 Hz, H-11), 2.79 (1*H*, m, H-3β), 1.03 (3*H*, s, H-25), 0.91(3*H*, s, H-23), 0.87 (3*H*, d, J= 6.5 Hz, H-29), 0.85 (3*H*, s, H-24), 0.81 (3*H*, d, J= 6.5 Hz, H-30), 0.79 (3*H*, s, H-26), 0.75 (3*H*, s, H-27), 0.74 (3*H*, s, H-28); 13 C NMR (100 MHz, CDCl3) δ 36.1(C-1), 21.5(C-2), 86.1(C-3), 38.5(C-4), 47.4(C-5), 20.6(C-6), 26.7(C-7), 41.3(C-8), 149.3(C-9), 39.8(C-10), 114.0(C-11), 36.2(C-12), 36.9(C-13), 38.3(C-14), 29.8(C-15), 36.2(C-16), 43.1(C-17), 52.3(C-18), 20.4(C-19), 28.6(C-20), 59.9(C-21), 31.0(C-22), 28.5(C-23), 22.3(C-24), 22.2(C-25), 17.3(C-26), 15.5(C-27), 14.2(C-28), 23.2(C-29), 22.1(C-30), 57.5(–OCH3). CD CH3CN); λmax/nm(Δε): 186(-0.70), 199(1.76), 215(0.58), 228(-0.14), 256(0.18), 299(-0.20).

### Refinement

H atoms were treated as riding in idealized positions, with C—H distances in the range 0.95–1.00 Å, depending on the atom type. Displacement parameters for H atoms were assigned as *U*_iso_ = 1.2Ueq of the attached atom (1.5 for methyl groups).

### Crystal data

**Table:**

C_31_H_52_O	*Z* = 1
*M**_r_* = 440.73	*F*(000) = 246
Triclinic, *P*1	*D*_x_ = 1.131 Mg m^−^^3^
Hall symbol: P 1	Cu *K*α radiation, λ = 1.5418 Å
*a* = 6.2684 (2) Å	Cell parameters from 11404 reflections
*b* = 7.1162 (3) Å	θ = 2.8–69.9°
*c* = 16.0814 (5) Å	µ = 0.48 mm^−^^1^
α = 96.812 (3)°	*T* = 120 K
β = 91.079 (3)°	Block, colourless
γ = 114.397 (4)°	0.60 × 0.50 × 0.40 mm
*V* = 646.86 (4) Å^3^	

### Data collection

**Table:**

Oxford Diffraction Xcalibur Eos Gemini diffractometer	4415 independent reflections
Radiation source: fine-focus sealed tube	4408 reflections with *I* > 2σ(*I*)
graphite	*R*_int_ = 0.011
Detector resolution: 16.0356 pixels mm^-1^	θ_max_ = 70.1°, θ_min_ = 2.8°
ω scans	*h* = −7→7
Absorption correction: multi-scan (*CrysAlis PRO*; Oxford Diffraction, 2010)	*k* = −8→8
*T*_min_ = 0.658, *T*_max_ = 1.0	*l* = −19→19
10247 measured reflections	

### Refinement

**Table:**

Refinement on *F*^2^	Secondary atom site location: difference Fourier map
Least-squares matrix: full	Hydrogen site location: inferred from neighbouring sites
*R*[*F*^2^ > 2σ(*F*^2^)] = 0.036	H-atom parameters constrained
*wR*(*F*^2^) = 0.096	*w* = 1/[σ^2^(*F*_o_^2^) + (0.0685*P*)^2^ + 0.1188*P*] where *P* = (*F*_o_^2^ + 2*F*_c_^2^)/3
*S* = 1.04	(Δ/σ)_max_ < 0.001
4415 reflections	Δρ_max_ = 0.27 e Å^−^^3^
298 parameters	Δρ_min_ = −0.18 e Å^−^^3^
3 restraints	Absolute structure: Flack (1983), 1952 Friedel pairs
Primary atom site location: structure-invariant direct methods	Flack parameter: 0.02 (22)

### Special details

**Table:**

Geometry. All e.s.d.'s (except the e.s.d. in the dihedral angle between two l.s. planes) are estimated using the full covariance matrix. The cell e.s.d.'s are taken into account individually in the estimation of e.s.d.'s in distances, angles and torsion angles; correlations between e.s.d.'s in cell parameters are only used when they are defined by crystal symmetry. An approximate (isotropic) treatment of cell e.s.d.'s is used for estimating e.s.d.'s involving l.s. planes.
Refinement. Refinement of *F*^2^ against ALL reflections. The weighted *R*-factor *wR* and goodness of fit *S* are based on *F*^2^, conventional *R*-factors *R* are based on *F*, with *F* set to zero for negative *F*^2^. The threshold expression of *F*^2^ > σ(*F*^2^) is used only for calculating *R*-factors(gt) *etc*. and is not relevant to the choice of reflections for refinement. *R*-factors based on *F*^2^ are statistically about twice as large as those based on *F*, and *R*- factors based on ALL data will be even larger.

### Fractional atomic coordinates and isotropic or equivalent isotropic displacement parameters (Å^2^)

**Table:**

	*x*	*y*	*z*	*U*_iso_*/*U*_eq_	
C1	0.5138 (3)	−0.3358 (2)	0.40741 (9)	0.0184 (3)	
H1B	0.5050	−0.4577	0.4335	0.022*	
H1A	0.3552	−0.3370	0.4057	0.022*	
C2	0.5864 (3)	−0.3558 (2)	0.31749 (9)	0.0198 (3)	
H2B	0.7412	−0.3622	0.3188	0.024*	
H2A	0.4706	−0.4871	0.2852	0.024*	
C3	0.6011 (3)	−0.1728 (2)	0.27380 (9)	0.0192 (3)	
H3	0.6561	−0.1893	0.2165	0.023*	
C4	0.7721 (3)	0.0390 (2)	0.32172 (8)	0.0179 (3)	
C5	0.7148 (2)	0.0538 (2)	0.41576 (8)	0.0152 (3)	
H5	0.5571	0.0575	0.4149	0.018*	
C6	0.8804 (3)	0.2588 (2)	0.46836 (9)	0.0197 (3)	
H6B	1.0362	0.2578	0.4785	0.024*	
H6A	0.9012	0.3767	0.4374	0.024*	
C7	0.7799 (3)	0.2871 (2)	0.55207 (9)	0.0200 (3)	
H7A	0.6303	0.2993	0.5415	0.024*	
H7B	0.8907	0.4186	0.5857	0.024*	
C8	0.7348 (2)	0.1057 (2)	0.60306 (8)	0.0153 (3)	
H8	0.8920	0.1098	0.6188	0.018*	
C9	0.5957 (2)	−0.1040 (2)	0.54887 (8)	0.0150 (3)	
C10	0.6864 (2)	−0.1349 (2)	0.46198 (8)	0.0154 (3)	
C11	0.4202 (3)	−0.2573 (2)	0.57888 (9)	0.0197 (3)	
H11	0.3307	−0.3797	0.5410	0.024*	
C12	0.3540 (3)	−0.2501 (2)	0.66850 (9)	0.0199 (3)	
H12B	0.3205	−0.3859	0.6878	0.024*	
H12A	0.2094	−0.2262	0.6713	0.024*	
C13	0.5521 (2)	−0.0759 (2)	0.72699 (8)	0.0154 (3)	
C14	0.6215 (2)	0.1294 (2)	0.68650 (8)	0.0145 (3)	
C15	0.7946 (3)	0.3152 (2)	0.74854 (8)	0.0179 (3)	
H15B	0.9407	0.2963	0.7576	0.021*	
H15A	0.8356	0.4446	0.7231	0.021*	
C16	0.6969 (3)	0.3411 (2)	0.83427 (9)	0.0191 (3)	
H16B	0.8175	0.4612	0.8710	0.023*	
H16A	0.5581	0.3716	0.8261	0.023*	
C17	0.6263 (2)	0.1459 (2)	0.87773 (8)	0.0173 (3)	
C18	0.4636 (2)	−0.0441 (2)	0.81386 (8)	0.0166 (3)	
H18	0.3244	−0.0145	0.8016	0.020*	
C19	0.3707 (3)	−0.2239 (2)	0.86612 (10)	0.0259 (3)	
H19B	0.4854	−0.2846	0.8728	0.031*	
H19A	0.2198	−0.3348	0.8404	0.031*	
C20	0.3376 (3)	−0.1160 (3)	0.95141 (10)	0.0285 (4)	
H20B	0.4129	−0.1513	0.9982	0.034*	
H20A	0.1684	−0.1626	0.9600	0.034*	
C21	0.4542 (3)	0.1223 (2)	0.94855 (9)	0.0192 (3)	
H21	0.3286	0.1607	0.9274	0.023*	
C22	0.5449 (3)	0.2481 (3)	1.03637 (9)	0.0248 (3)	
H22	0.6626	0.2042	1.0605	0.030*	
C23	0.7374 (3)	0.2111 (2)	0.28168 (9)	0.0264 (3)	
H23A	0.7427	0.1863	0.2206	0.040*	
H23C	0.8627	0.3469	0.3041	0.040*	
H23B	0.5848	0.2097	0.2947	0.040*	
C24	1.0260 (3)	0.0673 (2)	0.30911 (10)	0.0243 (3)	
H24C	1.0491	−0.0499	0.3275	0.036*	
H24B	1.1358	0.1977	0.3423	0.036*	
H24A	1.0547	0.0721	0.2495	0.036*	
C25	0.9183 (3)	−0.1575 (2)	0.47981 (9)	0.0200 (3)	
H25C	0.8942	−0.2552	0.5205	0.030*	
H25B	1.0420	−0.0212	0.5027	0.030*	
H25A	0.9650	−0.2102	0.4274	0.030*	
C26	0.7569 (3)	−0.1429 (2)	0.73227 (9)	0.0205 (3)	
H26C	0.7243	−0.2442	0.7719	0.031*	
H26B	0.9031	−0.0205	0.7516	0.031*	
H26A	0.7732	−0.2064	0.6766	0.031*	
C27	0.4046 (3)	0.1723 (2)	0.66699 (9)	0.0192 (3)	
H27B	0.4565	0.3150	0.6540	0.029*	
H27C	0.3109	0.1553	0.7159	0.029*	
H27A	0.3091	0.0738	0.6187	0.029*	
C28	0.8488 (3)	0.1317 (3)	0.91300 (10)	0.0259 (3)	
H28B	0.8048	−0.0020	0.9342	0.039*	
H28C	0.9271	0.2459	0.9589	0.039*	
H28A	0.9557	0.1421	0.8683	0.039*	
C29	0.3424 (3)	0.1982 (3)	1.09390 (10)	0.0299 (4)	
H29A	0.2243	0.2392	1.0712	0.045*	
H29C	0.4025	0.2751	1.1502	0.045*	
H29B	0.2708	0.0483	1.0971	0.045*	
C30	0.6642 (4)	0.4834 (3)	1.03546 (11)	0.0385 (4)	
H30B	0.8099	0.5187	1.0070	0.058*	
H30C	0.7004	0.5551	1.0933	0.058*	
H30A	0.5587	0.5273	1.0055	0.058*	
C31	0.2276 (3)	−0.3154 (3)	0.19751 (10)	0.0283 (4)	
H31B	0.3059	−0.2873	0.1453	0.043*	
H31A	0.0790	−0.3013	0.1929	0.043*	
H31C	0.1965	−0.4573	0.2075	0.043*	
O1	0.37357 (19)	−0.17199 (17)	0.26493 (7)	0.0247 (2)	

### Atomic displacement parameters (Å^2^)

**Table:**

	*U*^11^	*U*^22^	*U*^33^	*U*^12^	*U*^13^	*U*^23^
C1	0.0232 (8)	0.0137 (7)	0.0171 (7)	0.0071 (6)	0.0013 (6)	0.0005 (5)
C2	0.0255 (8)	0.0165 (7)	0.0180 (7)	0.0104 (6)	0.0000 (6)	−0.0020 (5)
C3	0.0238 (8)	0.0222 (7)	0.0143 (6)	0.0132 (6)	0.0022 (5)	−0.0010 (5)
C4	0.0239 (8)	0.0176 (7)	0.0139 (6)	0.0100 (6)	0.0027 (5)	0.0027 (5)
C5	0.0163 (7)	0.0152 (6)	0.0149 (6)	0.0074 (6)	0.0032 (5)	0.0016 (5)
C6	0.0249 (8)	0.0139 (7)	0.0173 (7)	0.0047 (6)	0.0042 (6)	0.0031 (5)
C7	0.0280 (8)	0.0116 (6)	0.0167 (6)	0.0048 (6)	0.0048 (6)	0.0012 (5)
C8	0.0167 (7)	0.0137 (6)	0.0143 (6)	0.0053 (5)	0.0009 (5)	0.0023 (5)
C9	0.0170 (7)	0.0135 (6)	0.0149 (6)	0.0071 (6)	0.0000 (5)	0.0010 (5)
C10	0.0156 (7)	0.0140 (6)	0.0167 (6)	0.0066 (6)	0.0008 (5)	0.0011 (5)
C11	0.0210 (7)	0.0151 (7)	0.0174 (6)	0.0031 (6)	0.0015 (6)	−0.0021 (5)
C12	0.0193 (7)	0.0153 (6)	0.0196 (7)	0.0019 (6)	0.0041 (6)	0.0010 (5)
C13	0.0143 (7)	0.0143 (6)	0.0166 (6)	0.0048 (6)	0.0019 (5)	0.0025 (5)
C14	0.0141 (7)	0.0129 (6)	0.0151 (6)	0.0043 (5)	0.0013 (5)	0.0013 (5)
C15	0.0179 (7)	0.0147 (7)	0.0164 (7)	0.0028 (6)	0.0030 (5)	0.0001 (5)
C16	0.0183 (7)	0.0186 (7)	0.0163 (6)	0.0047 (6)	0.0012 (5)	−0.0019 (5)
C17	0.0151 (7)	0.0225 (7)	0.0139 (6)	0.0082 (6)	0.0002 (5)	0.0000 (5)
C18	0.0151 (7)	0.0181 (7)	0.0165 (7)	0.0067 (6)	0.0022 (5)	0.0023 (5)
C19	0.0324 (9)	0.0232 (7)	0.0217 (7)	0.0101 (7)	0.0095 (6)	0.0054 (6)
C20	0.0356 (9)	0.0290 (8)	0.0215 (7)	0.0128 (7)	0.0106 (7)	0.0067 (6)
C21	0.0181 (7)	0.0262 (8)	0.0145 (6)	0.0106 (6)	0.0008 (5)	0.0021 (5)
C22	0.0242 (8)	0.0374 (9)	0.0151 (6)	0.0163 (7)	−0.0018 (6)	−0.0004 (6)
C23	0.0421 (10)	0.0224 (7)	0.0180 (7)	0.0163 (7)	0.0038 (6)	0.0044 (6)
C24	0.0250 (8)	0.0253 (7)	0.0216 (7)	0.0095 (6)	0.0091 (6)	0.0026 (6)
C25	0.0231 (7)	0.0236 (7)	0.0179 (6)	0.0146 (6)	0.0004 (5)	0.0024 (5)
C26	0.0258 (8)	0.0215 (7)	0.0191 (6)	0.0139 (6)	0.0050 (6)	0.0056 (5)
C27	0.0205 (7)	0.0231 (7)	0.0173 (6)	0.0120 (6)	0.0024 (5)	0.0034 (5)
C28	0.0208 (8)	0.0415 (9)	0.0191 (7)	0.0172 (7)	−0.0007 (6)	0.0021 (6)
C29	0.0354 (9)	0.0415 (9)	0.0166 (7)	0.0205 (8)	0.0048 (6)	0.0015 (6)
C30	0.0439 (11)	0.0391 (10)	0.0198 (8)	0.0078 (8)	0.0042 (7)	−0.0077 (7)
C31	0.0282 (9)	0.0290 (8)	0.0236 (7)	0.0094 (7)	−0.0047 (6)	−0.0015 (6)
O1	0.0263 (6)	0.0291 (6)	0.0206 (5)	0.0161 (5)	−0.0056 (4)	−0.0052 (4)

### Geometric parameters (Å, °)

**Table:**

C1—C2	1.5324 (19)	C7—H7A	0.9900
C1—C10	1.5405 (18)	C7—H7B	0.9900
C2—C3	1.522 (2)	C8—H8	1.0000
C3—O1	1.4333 (17)	C11—H11	0.9500
C3—C4	1.541 (2)	C12—H12B	0.9900
C4—C23	1.5374 (19)	C12—H12A	0.9900
C4—C24	1.540 (2)	C15—H15B	0.9900
C4—C5	1.5625 (18)	C15—H15A	0.9900
C5—C6	1.5310 (18)	C16—H16B	0.9900
C5—C10	1.5596 (18)	C16—H16A	0.9900
C6—C7	1.5238 (19)	C18—H18	1.0000
C7—C8	1.5420 (18)	C19—H19B	0.9900
C8—C9	1.5276 (17)	C19—H19A	0.9900
C8—C14	1.5540 (18)	C20—H20B	0.9900
C9—C11	1.337 (2)	C20—H20A	0.9900
C9—C10	1.5441 (18)	C21—H21	1.0000
C10—C25	1.5518 (18)	C22—H22	1.0000
C11—C12	1.5081 (19)	C23—H23A	0.9800
C12—C13	1.5398 (19)	C23—H23C	0.9800
C13—C18	1.5389 (18)	C23—H23B	0.9800
C13—C26	1.5475 (18)	C24—H24C	0.9800
C13—C14	1.5681 (17)	C24—H24B	0.9800
C14—C15	1.5433 (18)	C24—H24A	0.9800
C14—C27	1.5459 (18)	C25—H25C	0.9800
C15—C16	1.5408 (18)	C25—H25B	0.9800
C16—C17	1.532 (2)	C25—H25A	0.9800
C17—C28	1.5416 (19)	C26—H26C	0.9800
C17—C18	1.5542 (18)	C26—H26B	0.9800
C17—C21	1.559 (2)	C26—H26A	0.9800
C18—C19	1.530 (2)	C27—H27B	0.9800
C19—C20	1.551 (2)	C27—H27C	0.9800
C20—C21	1.552 (2)	C27—H27A	0.9800
C21—C22	1.5389 (18)	C28—H28B	0.9800
C22—C30	1.528 (3)	C28—H28C	0.9800
C22—C29	1.531 (2)	C28—H28A	0.9800
C31—O1	1.4081 (17)	C29—H29A	0.9800
C1—H1B	0.9900	C29—H29C	0.9800
C1—H1A	0.9900	C29—H29B	0.9800
C2—H2B	0.9900	C30—H30B	0.9800
C2—H2A	0.9900	C30—H30C	0.9800
C3—H3	1.0000	C30—H30A	0.9800
C5—H5	1.0000	C31—H31B	0.9800
C6—H6B	0.9900	C31—H31A	0.9800
C6—H6A	0.9900	C31—H31C	0.9800
			
C2—C1—C10	112.49 (11)	C9—C11—H11	117.6
C3—C2—C1	111.47 (11)	C12—C11—H11	117.6
O1—C3—C2	110.02 (12)	C11—C12—H12B	109.4
O1—C3—C4	108.37 (10)	C13—C12—H12B	109.4
C2—C3—C4	112.71 (11)	C11—C12—H12A	109.4
C23—C4—C24	107.02 (13)	C13—C12—H12A	109.4
C23—C4—C3	107.93 (11)	H12B—C12—H12A	108.0
C24—C4—C3	108.99 (11)	C16—C15—H15B	108.8
C23—C4—C5	109.08 (11)	C14—C15—H15B	108.8
C24—C4—C5	113.95 (11)	C16—C15—H15A	108.8
C3—C4—C5	109.68 (11)	C14—C15—H15A	108.8
C6—C5—C10	110.54 (10)	H15B—C15—H15A	107.7
C6—C5—C4	113.02 (11)	C17—C16—H16B	109.2
C10—C5—C4	116.75 (10)	C15—C16—H16B	109.2
C7—C6—C5	110.12 (12)	C17—C16—H16A	109.2
C6—C7—C8	112.85 (11)	C15—C16—H16A	109.2
C9—C8—C7	110.87 (10)	H16B—C16—H16A	107.9
C9—C8—C14	112.63 (11)	C19—C18—H18	104.2
C7—C8—C14	112.86 (10)	C13—C18—H18	104.2
C11—C9—C8	121.21 (12)	C17—C18—H18	104.2
C11—C9—C10	122.38 (11)	C18—C19—H19B	111.3
C8—C9—C10	116.10 (11)	C20—C19—H19B	111.3
C1—C10—C9	111.82 (11)	C18—C19—H19A	111.3
C1—C10—C25	108.20 (11)	C20—C19—H19A	111.3
C9—C10—C25	105.67 (10)	H19B—C19—H19A	109.2
C1—C10—C5	108.28 (10)	C19—C20—H20B	110.3
C9—C10—C5	108.41 (10)	C21—C20—H20B	110.3
C25—C10—C5	114.52 (11)	C19—C20—H20A	110.3
C9—C11—C12	124.77 (12)	C21—C20—H20A	110.3
C11—C12—C13	111.37 (12)	H20B—C20—H20A	108.5
C18—C13—C12	110.26 (11)	C22—C21—H21	106.7
C18—C13—C26	111.86 (11)	C20—C21—H21	106.7
C12—C13—C26	106.74 (11)	C17—C21—H21	106.7
C18—C13—C14	108.59 (10)	C30—C22—H22	108.1
C12—C13—C14	107.04 (10)	C29—C22—H22	108.1
C26—C13—C14	112.23 (11)	C21—C22—H22	108.1
C15—C14—C27	107.89 (10)	C4—C23—H23A	109.5
C15—C14—C8	111.00 (11)	C4—C23—H23C	109.5
C27—C14—C8	108.65 (10)	H23A—C23—H23C	109.5
C15—C14—C13	109.22 (10)	C4—C23—H23B	109.5
C27—C14—C13	111.49 (11)	H23A—C23—H23B	109.5
C8—C14—C13	108.61 (10)	H23C—C23—H23B	109.5
C16—C15—C14	113.60 (11)	C4—C24—H24C	109.5
C17—C16—C15	112.21 (11)	C4—C24—H24B	109.5
C16—C17—C28	109.54 (13)	H24C—C24—H24B	109.5
C16—C17—C18	107.77 (10)	C4—C24—H24A	109.5
C28—C17—C18	115.07 (11)	H24C—C24—H24A	109.5
C16—C17—C21	116.94 (11)	H24B—C24—H24A	109.5
C28—C17—C21	109.01 (11)	C10—C25—H25C	109.5
C18—C17—C21	98.36 (11)	C10—C25—H25B	109.5
C19—C18—C13	120.14 (11)	H25C—C25—H25B	109.5
C19—C18—C17	104.13 (11)	C10—C25—H25A	109.5
C13—C18—C17	117.98 (11)	H25C—C25—H25A	109.5
C18—C19—C20	102.56 (12)	H25B—C25—H25A	109.5
C19—C20—C21	107.16 (12)	C13—C26—H26C	109.5
C22—C21—C20	112.19 (12)	C13—C26—H26B	109.5
C22—C21—C17	120.19 (12)	H26C—C26—H26B	109.5
C20—C21—C17	103.53 (11)	C13—C26—H26A	109.5
C30—C22—C29	109.14 (13)	H26C—C26—H26A	109.5
C30—C22—C21	113.42 (13)	H26B—C26—H26A	109.5
C29—C22—C21	109.97 (13)	C14—C27—H27B	109.5
C31—O1—C3	113.55 (11)	C14—C27—H27C	109.5
C2—C1—H1B	109.1	H27B—C27—H27C	109.5
C10—C1—H1B	109.1	C14—C27—H27A	109.5
C2—C1—H1A	109.1	H27B—C27—H27A	109.5
C10—C1—H1A	109.1	H27C—C27—H27A	109.5
H1B—C1—H1A	107.8	C17—C28—H28B	109.5
C3—C2—H2B	109.3	C17—C28—H28C	109.5
C1—C2—H2B	109.3	H28B—C28—H28C	109.5
C3—C2—H2A	109.3	C17—C28—H28A	109.5
C1—C2—H2A	109.3	H28B—C28—H28A	109.5
H2B—C2—H2A	108.0	H28C—C28—H28A	109.5
O1—C3—H3	108.6	C22—C29—H29A	109.5
C2—C3—H3	108.6	C22—C29—H29C	109.5
C4—C3—H3	108.6	H29A—C29—H29C	109.5
C6—C5—H5	105.1	C22—C29—H29B	109.5
C10—C5—H5	105.1	H29A—C29—H29B	109.5
C4—C5—H5	105.1	H29C—C29—H29B	109.5
C7—C6—H6B	109.6	C22—C30—H30B	109.5
C5—C6—H6B	109.6	C22—C30—H30C	109.5
C7—C6—H6A	109.6	H30B—C30—H30C	109.5
C5—C6—H6A	109.6	C22—C30—H30A	109.5
H6B—C6—H6A	108.2	H30B—C30—H30A	109.5
C6—C7—H7A	109.0	H30C—C30—H30A	109.5
C8—C7—H7A	109.0	O1—C31—H31B	109.5
C6—C7—H7B	109.0	O1—C31—H31A	109.5
C8—C7—H7B	109.0	H31B—C31—H31A	109.5
H7A—C7—H7B	107.8	O1—C31—H31C	109.5
C9—C8—H8	106.7	H31B—C31—H31C	109.5
C7—C8—H8	106.7	H31A—C31—H31C	109.5
C14—C8—H8	106.7		
			
C10—C1—C2—C3	−59.20 (15)	C7—C8—C14—C27	50.92 (14)
C1—C2—C3—O1	−63.96 (15)	C9—C8—C14—C13	45.87 (13)
C1—C2—C3—C4	57.11 (16)	C7—C8—C14—C13	172.36 (11)
O1—C3—C4—C23	−47.33 (15)	C18—C13—C14—C15	53.03 (13)
C2—C3—C4—C23	−169.34 (12)	C12—C13—C14—C15	172.05 (11)
O1—C3—C4—C24	−163.22 (11)	C26—C13—C14—C15	−71.16 (13)
C2—C3—C4—C24	74.77 (15)	C18—C13—C14—C27	−66.11 (13)
O1—C3—C4—C5	71.40 (13)	C12—C13—C14—C27	52.92 (13)
C2—C3—C4—C5	−50.61 (14)	C26—C13—C14—C27	169.71 (11)
C23—C4—C5—C6	−63.07 (15)	C18—C13—C14—C8	174.21 (10)
C24—C4—C5—C6	56.44 (15)	C12—C13—C14—C8	−66.76 (12)
C3—C4—C5—C6	178.92 (11)	C26—C13—C14—C8	50.03 (13)
C23—C4—C5—C10	167.06 (12)	C27—C14—C15—C16	63.76 (14)
C24—C4—C5—C10	−73.43 (15)	C8—C14—C15—C16	−177.30 (10)
C3—C4—C5—C10	49.05 (15)	C13—C14—C15—C16	−57.58 (14)
C10—C5—C6—C7	−61.27 (14)	C14—C15—C16—C17	57.96 (15)
C4—C5—C6—C7	165.77 (11)	C15—C16—C17—C28	74.67 (14)
C5—C6—C7—C8	57.48 (15)	C15—C16—C17—C18	−51.19 (14)
C6—C7—C8—C9	−49.90 (16)	C15—C16—C17—C21	−160.71 (12)
C6—C7—C8—C14	−177.32 (11)	C12—C13—C18—C19	59.69 (16)
C7—C8—C9—C11	−137.79 (14)	C26—C13—C18—C19	−58.92 (17)
C14—C8—C9—C11	−10.25 (17)	C14—C13—C18—C19	176.68 (12)
C7—C8—C9—C10	48.41 (15)	C12—C13—C18—C17	−171.39 (11)
C14—C8—C9—C10	175.96 (10)	C26—C13—C18—C17	69.99 (15)
C2—C1—C10—C9	173.16 (10)	C14—C13—C18—C17	−54.41 (14)
C2—C1—C10—C25	−70.88 (14)	C16—C17—C18—C19	−171.12 (12)
C2—C1—C10—C5	53.79 (14)	C28—C17—C18—C19	66.36 (15)
C11—C9—C10—C1	15.03 (17)	C21—C17—C18—C19	−49.26 (12)
C8—C9—C10—C1	−171.26 (11)	C16—C17—C18—C13	52.82 (14)
C11—C9—C10—C25	−102.47 (15)	C28—C17—C18—C13	−69.71 (16)
C8—C9—C10—C25	71.24 (13)	C21—C17—C18—C13	174.68 (11)
C11—C9—C10—C5	134.32 (14)	C13—C18—C19—C20	171.98 (12)
C8—C9—C10—C5	−51.97 (14)	C17—C18—C19—C20	37.10 (15)
C6—C5—C10—C1	178.59 (11)	C18—C19—C20—C21	−10.02 (17)
C4—C5—C10—C1	−50.38 (14)	C19—C20—C21—C22	−151.34 (13)
C6—C5—C10—C9	57.09 (14)	C19—C20—C21—C17	−20.29 (16)
C4—C5—C10—C9	−171.88 (11)	C16—C17—C21—C22	−77.55 (16)
C6—C5—C10—C25	−60.60 (14)	C28—C17—C21—C22	47.34 (17)
C4—C5—C10—C25	70.43 (15)	C18—C17—C21—C22	167.58 (12)
C8—C9—C11—C12	−5.5 (2)	C16—C17—C21—C20	156.35 (12)
C10—C9—C11—C12	167.90 (13)	C28—C17—C21—C20	−78.77 (15)
C9—C11—C12—C13	−16.42 (19)	C18—C17—C21—C20	41.47 (13)
C11—C12—C13—C18	169.17 (11)	C20—C21—C22—C30	179.12 (14)
C11—C12—C13—C26	−69.12 (14)	C17—C21—C22—C30	57.15 (18)
C11—C12—C13—C14	51.23 (14)	C20—C21—C22—C29	−58.36 (16)
C9—C8—C14—C15	165.96 (10)	C17—C21—C22—C29	179.67 (13)
C7—C8—C14—C15	−67.56 (14)	C2—C3—O1—C31	−79.35 (14)
C9—C8—C14—C27	−75.56 (13)	C4—C3—O1—C31	157.01 (12)
